# Supplementary material for: Causal Relationship between Aging and Anorexia Nervosa: A White-Matter-Microstructure-Mediated Mendelian Randomization Analysis
Source: Biomedicines. 2024 Aug 16;12(8):1874. doi: 10.3390/biomedicines12081874 (PMC11351342; doi:10.3390/biomedicines12081874)
Supplement: Supplementary file 1 [file biomedicines-12-01874-s001.zip › Supplementary Figure/Figure S4/Singe SNP plot of MR analysis for TL to ubm-b-1482.pdf]

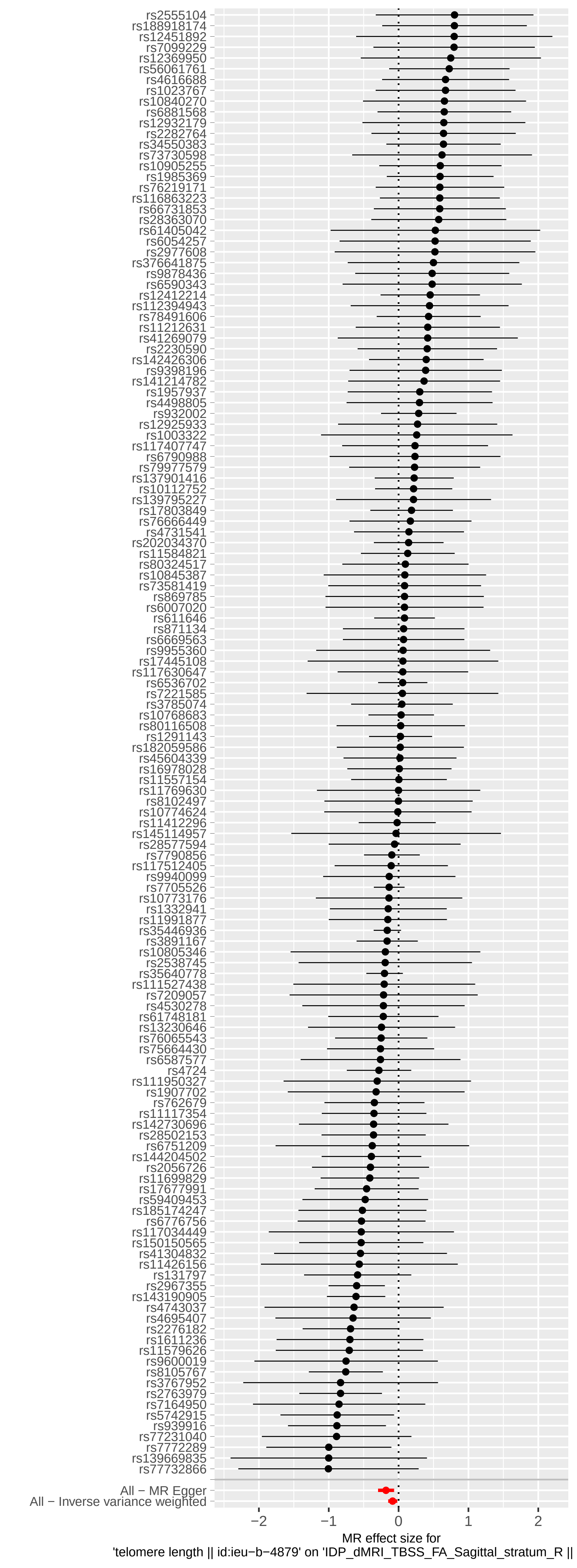

rs2555104
rs188918174
rs12451892
rs7099229
rs12369950
rs56061761
rs4616688
rs1023767
rs10840270
rs6881568
rs12932179
rs2282764
rs34550383
rs73730598
rs10905255
rs1985369
rs76219171
rs116863223
rs66731853
rs28363070
rs61405042
rs6054257
rs2977608
rs376641875
rs9878436
rs6590343
rs12412214
rs112394943
rs78491606
rs11212631
rs41269079
rs2230590
rs142426306
rs9398196
rs141214782
rs1957937
rs4498805
rs932002
rs12925933
rs1003322
rs117407747
rs6790988
rs79977579
rs137901416
rs10112752
rs139795227
rs17803849
rs76666449
rs4731541
rs202034370
rs11584821
rs80324517
rs10845387
rs73581419
rs869785
rs6007020
rs611646
rs871134
rs6669563
rs9955360
rs17445108
rs117630647
rs6536702
rs7221585
rs3785074
rs10768683
rs80116508
rs1291143
rs182059586
rs45604339
rs16978028
rs11557154
rs11769630
rs8102497
rs10774624
rs11412296
rs145114957
rs28577594
rs7790856
rs117512405
rs9940099
rs7705526
rs10773176
rs1332941
rs11991877
rs35446936
rs3891167
rs10805346
rs2538745
rs35640778
rs111527438
rs7209057
rs4530278
rs61748181
rs13230646
rs76065543
rs75664430
rs6587577
rs4724
rs111950327
rs1907702
rs762679
rs11117354
rs142730696
rs28502153
rs6751209
rs144204502
rs2056726
rs11699829
rs17677991
rs59409453
rs185174247
rs6776756
rs117034449
rs150150565
rs41304832
rs11426156
rs131797
rs2967355
rs143190905
rs4743037
rs4695407
rs2276182
rs1611236
rs11579626
rs9600019
rs8105767
rs3767952
rs2763979
rs7164950
rs5742915
rs939916
rs77231040
rs7772289
rs139669835
rs77732866
All − MR Egger
All − Inverse variance weighted
−2
−1
0
1
2
MR effect size for
'telomere length || id:ieu−b−4879' on 'IDP_dMRI_TBSS_FA_Sagittal_stratum_R ||
